# **M**others **U**nderstand **A**nd **C**an do it (MUAC): a comparison of mothers and community health workers determining mid-upper arm circumference in 103 children aged from 6 months to 5 years

**DOI:** 10.1186/s13690-015-0074-z

**Published:** 2015-06-01

**Authors:** Nikki Blackwell, Mark Myatt, Thierry Allafort-Duverger, Amour Balogoun, Almou Ibrahim, André Briend

**Affiliations:** Alliance for International Medical Action (ALIMA), Dakar, Senegal; Department of Critical Care, University of Queensland, Brisbane, Australia; Brixton Health, Llawryglyn, Wales UK; Bien Être de la Femme et de l’Enfant (BEFEN), Niamey, Niger; Department for International Health, University of Tampere School of Medicine, Tampere, Finland; Department of Nutrition, Exercise and Sports, Faculty of Science, University of Copenhagen, Copenhagen, Denmark

**Keywords:** Severe acute malnutrition, Mid-upper arm circumference, Community management of acute malnutrition, Screening by mothers

## Abstract

**Background:**

Mid-upper arm circumference (MUAC) was recently endorsed and recommended for screening for acute malnutrition in the community. The objective of this study was to determine whether a colour-banded MUAC strap would allow minimally trained mothers to screen their own children for malnutrition, without locating the mid-point of the left upper arm by measurement, as currently recommended.

**Methods:**

A non-randomised non-blinded evaluation of mothers’ performance when measuring MUAC after minimal training, compared with trained Community Health Workers (CHW) following current MUAC protocols. The study was conducted in 2 villages in Mirriah, Zinder region, Niger where mothers classified one of their children (*n* = 103) aged 6–59 months (the current age range for admission into community malnutrition programs) using the MUAC tape.

**Results:**

Mothers’ had a sensitivity and specificity for classification of their child’s nutritional status of > 90% and > 80% respectively for global acute malnutrition (GAM, defined by a MUAC < 125 mm) and > 73% and > 98% for severe acute malnutrition (SAM, defined by a MUAC < 115 mm). The few children misclassified as not having SAM, were classified as having moderate acute malnutrition (MAM). The choice of arm did not influence the classification results; weighted Kappa of 0.88 for mothers and 0.91 for CHW represent almost perfect agreement. Errors occurred at the class boundaries and no gross errors were made.

**Conclusions:**

Advanced SAM is associated with severe complications, which often require hospital admission or cause death. Mothers (with MUAC tapes costing $0.06) can screen their children frequently allowing early diagnosis and treatment thereby becoming the focal point in scaling-up community management of acute malnutrition.

**Trial registration:**

The trial is registered with clinicaltrials.gov (Trial number NCT01790815)

## Background

United Nations Children’s Fund (UNICEF) and World Health Organisation (WHO specify any one of three independent criteria for defining severe acute malnutrition (SAM): the presence of bilateral pitting oedema, a weight-for-height z-score (WHZ) of less than −3, based on the WHO growth curves [1], or a MUAC of less than 115 mm. To define moderate acute malnutrition (MAM), a WHZ of less than −2 and greater than −3, or a MUAC of less than 125 mm and greater than 115 mm is used by many programmes.

Measures of weight-for-height have been used for many years for the diagnosis of SAM. MUAC was not widely recommended until the scaling up of community management of acute malnutrition (CMAM) occurred [2]. MUAC [3] is the best predictor of mortality in community malnutrition programmes [4]. Frequent screening gives the best results [5], the MUAC tape is cheap and simple; this might allow mothers to test their own children on a regular basis, and ensure they seek treatment in a CMAM program in a timely manner.

Community based management of SAM often relies on Community Health Workers (CHW), who may be unpaid volunteers, to conduct the anthropometric measurements of children being screened and treated for SAM. Currently this requires training, standardisation testing, and follow-up, motivation and retention of these staff may also be problematic. In training to perform a MUAC measurement, a CHW must learn to use the left not the right arm, and to measure and mark the mid-point of the upper-arm, between shoulder-tip and elbow [6]. The left upper arm has come to be used for MUAC measurement, because triceps and biceps muscle development may be asymmetric, being greater in the dominant arm than in the non-dominant arm (assumed to be the left). MUAC results may vary depending on where along the muscle belly of the biceps the tape is applied. While this may be relevant in adults who perform regular physical activity with their dominant arm – for example using a machete for harvesting, there is no evidence that these concerns apply to children under 5 years of age. Some earlier work around MUAC specified the use of the right arm rather than the left arm and did not specify measurement of the mid point of the upper arm [7], while a more recent study in Nigeria [8] did not specify which arm was to be used.

The hypothesis for this study is that mothers with minimal training can measure MUAC to classify the nutritional state of their children. This was initially confirmed in an in-patient setting with children admitted with SAM with complications (data not shown). The study hypothesis was tested in two villages in rural Niger. The rationale for teaching mothers to perform MUAC is to achieve an early diagnosis of SAM, which if acted upon in a timely manner would decrease mortality and morbidity related to malnutrition, reduce program costs due shorter treatment times and lowering the proportion of children requiring expensive in-patient care for SAM with complications (as shown in a study from Bangladesh [9,10]. If mothers are able to screen their own children for malnutrition with MUAC this may have implications for scaling up CMAM to optimise early diagnosis and intervention.

## Methods

This study was conducted according to the guidelines laid down in the Declaration of Helsinki. The study protocol was given ethical approval by the Mirriah Health District Ethics Committee, Niger (Approval Number: 22DS/MI/12). Verbal, as opposed to written, informed consent was obtained from all subjects because of limited literacy, consent was witnessed and formally recorded by another CHW acting as the scribe. The trial is registered with clinicaltrials.gov (Trial number NCT01790815).

**Figure 1:**
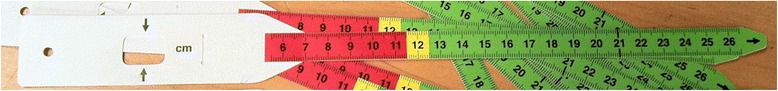
**MUAC tape ($0.06, UNICEF supply catalogue) showing 3 classes: red, yellow and green.**

The study was performed between September 2011 and April 2012, the community meeting places in 2 villages in Mirriah, a rural district in the Zinder region in southern Niger. There were 54 mothers with one of their children in the first village (Magema), and 49 mothers with one of their children in the second village (Berberkia). It was conducted by the Alliance of International Medical Action (ALIMA) and its local non-governmental organisation (NGO) partner BEFEN (Bien Etre de la Femme et de l’Enfant). UNICEF colour-coded and numbered MUAC tapes, calibrated in 1 mm gradations, were used for all phases of the study (Figure 1).

The manager of the CHW employed by BEFEN, who is well known in both villages, explained the purpose of the study to the village chiefs, elders and leaders. In each case an independent scribe was used, recording the consent and the colour class determined by the mother, and also noting the MUAC value in millimetres.

The planned study method had involved going from home to home, and performing a 5 minutes training session for each mother; however this proved impossible. In both villages, once the objective of showing mothers how to perform MUAC classifications had been explained to the village chief and other leaders, it was considered to be so important that they insisted on calling an immediate meeting of the villagers. Mothers and children formed a seated group around the investigators, circled by the older girls who stood to obtain a good view, with the men and boys standing behind. Each mother did one MUAC measurement on the left arm, followed directly by one MUAC measurement on the right arm of their child. The mother called out the colour classification (eg “Red”) and this was noted down by the investigator. This was then repeated by an experienced CHW testing the right and then the left arm of each child, having measured the midpoint according to current recommendations [6]. All the mothers in both villages wished to participate, however only one child from each mother was accepted into the study. Any child identified with SAM was referred to the local CMAM program (which was run by BEFEN).

In the statistical analysis, cross-tabulations were used to investigate the agreement between mothers’ results and the CHW reference measure. The Weighted Kappa was used as a numeric summary of inter-rater agreement between two raters or methods, beyond that which would be expected by chance. Kappa is a measure of agreement, standardized along a −1 to 1 scale, where positive numbers indicate agreement, with 1 being perfect agreement, negative numbers indicate disagreement, with minus one being total disagreement, and zero indicating agreement expected by chance [11,12]. The Weighted Kappa was used because of the ordinal nature of classifications (i.e. SAM is more serious than MAM, which is more serious than normal nutritional status). The weighting process treats gross misclassifications (e.g. true SAM classed as normal) as more important than misclassifications between adjacent classes. As a guide, the level of agreement demonstrated by Weighted Kappa values is: 0.0-0.2 ‘slight’; 0.21-0.40 ‘fair’, 0.41-0.60 ‘moderate’, 0.61-0.80 ‘substantial’ and 0.81-1.00 ‘almost perfect’ [13]. The mothers’ sensitivity, specificity, negative and positive predictive values (NPV and PPV) using the MUAC screening for SAM were calculated using the CHW measurements as the reference result. The *R* language was used [14] for data analysis and graphics.

## Results

Table 1 shows the ability of mothers to attribute correctly a MUAC colour class to their child, compared with the performance of CHW for SAM, in right and left arm.

**Table 1:** **Severe acute malnutrition, defined by a MUAC cut-off <115 mm: comparing classification by mothers and CHW, for each arm**

**Right arm**		**CHW**		**Left arm**		**CHW**	
	MUAC	<115 mm	≥115 mm		MUAC	<115 mm	≥115 mm
Mothers	<115 mm	12	3	Mothers	<115 mm	11	1
	≥115 mm	3	85		≥115 mm	4	87

Sensitivity = 80.0% (54.8%; 93.0%) Sensitivity = 73.3% (48.1%; 89.1%).

Specificity = 96.6% (90.5%, 98.8%) Specificity = 98.9% (93.8%; 99.8%).

PPV = 80.0% (54.8%; 93.0%) PPV = 91.7% (64.6%; 98.5%).

NPV = 96.6% (90.5%; 98.8%) NPV = 95.6% (89.2%; 98.3%).

Accuracy = 94.2% (87.9%; 97.3%) Accuracy = 95.6% (89.2%; 98.3%).

Table 2 shows the ability of mothers to attribute correctly a MUAC colour class for global acute malnutrition (GAM, defined as MUAC <125 mm) again in right and left arms. A mean difference was found between left and right arm measurements for mothers of 0.3 mm (95% CI: −0.19; 0.79), and for CHW 0.11 mm, (95% CI: −0.26; 0.47). These measurements did not differ significantly from zero (p = 0.22 and p = 0.56 respectively). The ratio of variances for left and right arm measurements for mothers (1.09) and CHW (1.05) did not differ significantly from one (p = 0.34 and p = 0.40) respectively.

**Table 2:** **Global acute malnutrition, defined by a MUAC cut-off <125 mm: comparing classification by mothers and CHW, for each arm**

**Right arm**	**CHW**			**Left arm**	**CHW**		
	MUAC	<125 mm	≥125 mm		MUAC	<125 mm	≥125 mm
Mothers	<125 mm	38	12	Mothers	<125 mm	39	11
	≥125 mm	2	51		≥125 mm	1	52

Sensitivity = 95.0% (83.5%; 98.6%) Sensitivity = 97.5% (87.1%; 99.6%).

Specificity = 81.0% (69.6%, 88.8%) Specificity = 82.5% (71.4%; 90.0%).

PPV = 76.0% (62.6%; 85.7%) PPV = 78.0% (64.8%; 87.3%).

NPV = 96.2% (87.3%; 99.0%) NPV = 98.1% (90.1%; 99.7%).

Accuracy = 86.4% (78.5%; 91.7%) Accuracy = 88.4% (80.7%; 93.2%).

When classification of both arms by mothers or by CHW was assessed using Weighted Kappa [11] (Tables 3 and 4), the observed agreement between classifications for right and left arm, was “almost perfect” for both arms [14].

**Table 3:** **Weighted Kappa results showing MUAC classification by mothers in each arm**

**Mothers**	**Class from left arm**			
**Class from right arm**		**SAM**	**MAM**	**Normal**
	SAM	12	3	0
	MAM	0	32	3
	Normal	0	3	50

Weighted Kappa = 0.88, (95% CI = 0.80, 0.96).

**Table 4:** **Weighted Kappa results showing MUAC classification by CHW in each arm**

**CHW**	**Class from left arm**			
**Class from right arm**		**SAM**	**MAM**	**Normal**
	SAM	14	1	0
	MAM	1	22	3
	Normal	0	2	60

Weighted Kappa 0.91, (95% CI = 0.84, 0.97).

The observed agreement in the ability of mother’s to classify their child using either arm, with the mid-point of the upper arm ascertained by eye, compared with the CHW classification with the mid-point of the left upper arm ascertained by measurement (ie the criteria by which a child is considered for entry into many CMAM programs) (Table 5) can be classified as “substantial” or better for both [13].

**Table 5:** **MUAC classification with mid-point of upper arm ascertained by eye by mothers, compared with the mid-point ascertained by measurement by CHW**

**Right arm**		**CHW**			**Left arm**		**CHW**		
		SAM	MAM	Normal			SAM	MAM	Normal
	SAM	12	3	0		SAM	11	1	0
Mothers	MAM	3	20	12	Mothers	MAM	4	23	11
	Normal	0	2	51		Normal	0	1	52

Weighted Kappa = 0.74, (95% CI = 0.63, 0.85) Weighted Kappa = 0.77, (95% CI =0.67, 0.87).

**Figure 2:**
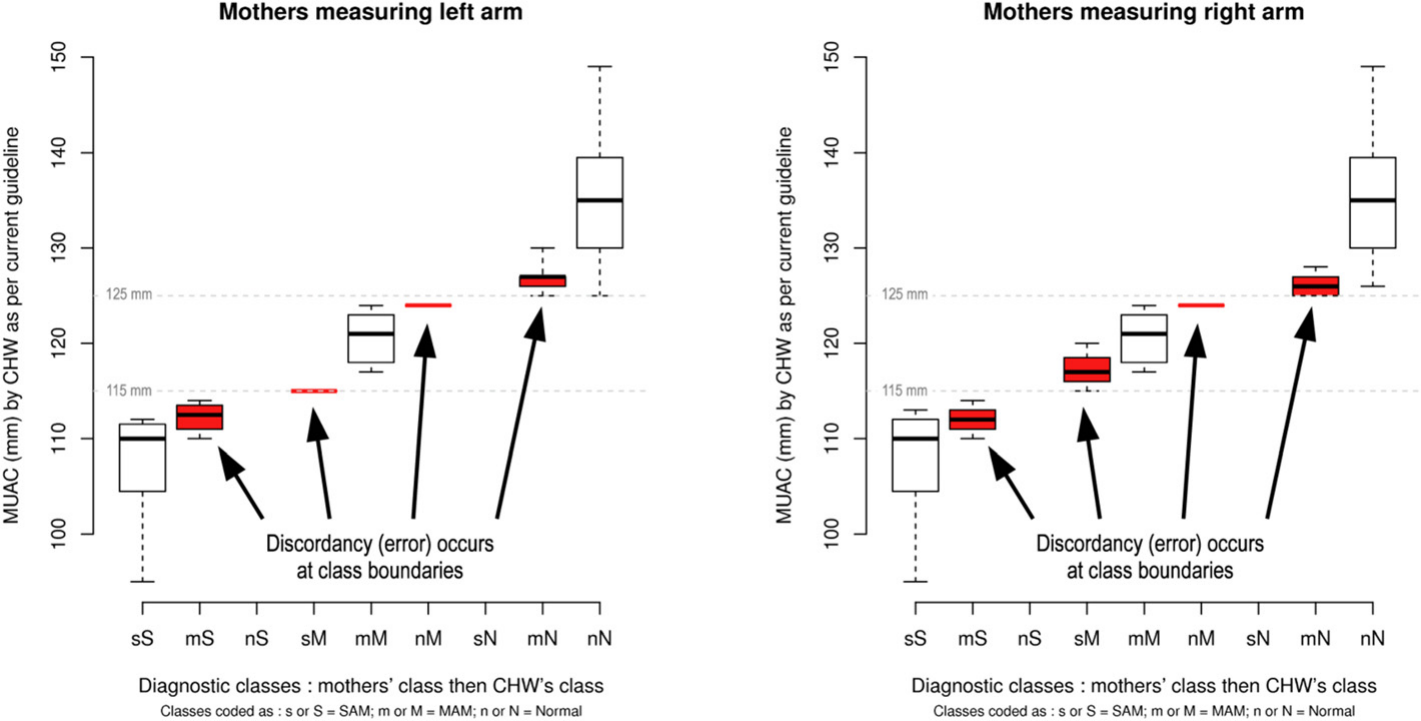
**MUAC classification errors, mothers compared with CHW, measuring the left arm, and MUAC classification errors, mothers compared with CHW, measuring the right arm.**

MUAC classification errors (or discordance) occurred at class boundaries, i.e. the border between red (SAM) and yellow (MAM), and yellow and green (normal), rather than randomly, and there are no gross discordances (Figure 2). Thus mothers do not classify a child as normal when the CHW diagnosis is SAM, nor do they classify a child as SAM when the CHW diagnosis is normal. MUAC testing in two villages, showed high levels of agreement regardless of which arm was used by mothers, as well as high agreement between readings by mothers and by CHW, with high case-finding sensitivity and specificity.

## Discussion

Almost 50 years after an early attempt to standardise the use of MUAC tapes, with trained health workers using the midpoint of the upper left arm [15], the present study shows mothers given minimal training can perform and interpret MUAC on young children in a village setting. It suggests that the choice of arm and exact location of the tape on the upper arm are not important for screening. This has implications for scaling up treatment to allow mass coverage and early intervention for SAM in vulnerable communities.

Importantly, all children misclassified as not having SAM, were nevertheless classified as MAM, none were considered ‘normal’, thus classification errors occurred at the classification boundaries. Classification by mothers measuring the mid point of the upper arm determined by eye, showed an accuracy of 86.4% – 88.4% in GAM, and 94.2% – 95.6% in SAM, compared with the current standard of the CHW classification. Roll-out of MUAC, done by mothers in the home would involve small group learning with each mother able to practice under supervision. This would be expected further to improve the quality of the screening. There was very low inter- and intra- observer variability, and high levels of agreement achieved between the current standard (results from CHW), compared with mothers with minimal training.

The study was conducted in free living village populations with a lower prevalence of SAM than a program setting. This is the environment where scaled up CMAM will occur, with mothers screening their own children for malnutrition. The prevalence of SAM in these 2 villages cannot be measured from these data, because only one child from each mother was entered into the study.

The Weighted Kappa values for inter-observer variation for the interpretation of classical physical signs elicited in clinical medicine are frequently less than 0.50. For example, raised respiratory rate (K = 0.25), central venous pressure (K = 0.3), bronchial breath sounds (K = 0.32) [16]. These errors reflect variations between clinicians, patients and the circumstances at the time of the examination. Such errors can be reduced by increased standardisation of the test method and by increasing the number of times the test is performed. The observed Kappa values are consistent with excellent agreement between mothers and CHW.

The first recommendation in a recent consultation paper comparing MUAC with WHZ [3], was for active case finding by MUAC to identify children requiring management of SAM at community level. MUAC certainly represents value for money, the MUAC tape being less than 1/1000 the cost of the equipment required to measure WHZ score.

Sadler and colleagues [9] in Bangladesh demonstrated that monthly MUAC screening for SAM performed by a network of female CHW can lead to very high program coverage. Timely case-finding and admission to a therapeutic feeding program resulted in a high cure rate (91.9%) and very low mortality (0.1%), with 92.5% achieving full adherence to the CMAM protocol and a low hospitalisation rate. In addition weight gain and length of stay were well within SPHERE minimum standards [17].

It is proposed that MUAC *class* rather than MUAC measurement is used by mothers for active case-finding in the home. This could be facilitated by the use of broader, colour-banded MUAC straps, as suggested recently [3]. Numberless colour-banded MUAC tapes have been available for many years [18]. One advantage of using a colour (red, yellow or green) for MUAC classification is that children of illiterate and/or innumerate mothers are not disadvantaged. Female literacy in the 15–24 year age group in Niger is estimated at 23% [19] although this is expected to be lower in rural areas.

The use of numbered MUAC tapes could only be required for monitoring response to treatment in therapeutic feeding programs but this approach needs validation. Numbered MUAC tapes may be useful as well in nutritional anthropometry surveys or surveillance systems that estimate prevalence of SAM using the PROBIT method [20].

Community leaders were keen for local women to learn the skill. In contrast men were not interested in learning the skill, while adamant that the women should learn. Mothers who had completed the task were observed to help other mothers perform the MUAC classification and the enthusiasm of girls (i.e. the next generation of mothers) to participate was striking. A future can be envisaged in which mothers in every home where childhood malnutrition is a risk are familiar with, and empowered to screen their children. Even in the most functional CMAM program, it is unusual for children to be screened more than once a month. Repeated screening with MUAC, if a mother is concerned about her child’s health, will increase the likelihood of early diagnosis, even if the initial screen classified the child incorrectly. Training mothers to perform MUAC could provide the most timely and proximal diagnosis of malnutrition in their young children. Furthermore programs could be designed to facilitate mothers monitoring their children once entered into a CMAM program, to reduce the number of repeat visits, often at great distance on foot, to the treatment centre, and increase retention in the program, although this approach has not yet been validated.

Advanced SAM is associated with complications, which often require hospital admission or cause death [9,10]. Earlier diagnosis, when the MUAC has just passed the threshold for intervention, should support prompt entry into treatment programs. This will lead to reduced mortality and length of stay, and favourable community impressions will lead to early treatment and in turn high cost-effectiveness.

Giving mothers more autonomy in monitoring the nutritional status of their children may provide the *tipping point* [21] in scaling up CMAM programmes. Many mothers and community leaders were delighted finally to comprehend how MUAC is used to make the diagnosis of SAM. They had witnessed malnutrition screening programs many times without understanding why one child was entered into a CMAM program while another was excluded.

In Niger, international and more recently national NGOs (eg BEFEN and Forum Santé Niger (FORSANI)) have been running nutrition programs for more than 8 years. This may explain why mothers and communities embraced the idea of doing MUAC themselves; this may not be the case in contexts unaccustomed to nutrition programs. Widespread dissemination of MUAC tapes may be an important step in raising community awareness of malnutrition [4].

Limitations of the study: mothers were chosen because the trial involved their village, thus they were not randomly selected. There are some missing data, which however do not change the overall highly significant results. Mothers classified first one arm then the second arm; this may have caused the results for each arm to be more alike than they were in reality.

It may be that this population was unusually receptive to learning to use MUAC tapes themselves, although only when the method was explained did the community recognize the treatment implications of the MUAC classifications. Perhaps more likely, this approach may illustrate the willingness of mothers to participate as fully as possible in promoting the health of their children.

The study reported here collected data on children under five years of age and the results may apply only to this age-group. The choice of arm on which MUAC is measured is likely to be important in adolescents and adults in whom the MUAC of the dominant arm is generally larger than the MUAC of the non-dominant arm. Some programs (e.g. supplementary feeding programs) admit both children aged under five years and adults (mostly pregnant and lactating women with a MUAC below a threshold between 210 mm and 230 mm). Training and supervision of program workers should continue to emphasise different procedures are used when measuring children and adults.

## Conclusions

This study suggests that with brief training, mothers from the Mirriah district of Niger were able to classify their children into one of the three colour classes on a MUAC tape. The study has demonstrated that it is not necessary to specify which arm is used nor formally to measure the middle of the upper-arm, further simplifying an already simple task. Asking mothers to determine only the colour class for their child, allows illiterate and/or innumerate women to perform this screening test on their children. Since there was no difference between the MUAC value between arms, or the mid point determined by measurement or by eye, CHW training in MUAC reading can be also simplified, allowing the use of either arm of the child, and estimating the half-way point by eye.

This study demonstrates that mothers can classify their children by MUAC, and it suggests MUAC done regularly by mothers should become the focal point for efforts to scale-up CMAM.

## Abbreviations

MUAC: Mid-upper arm circumference
CHW: Community Health Worker
CMAM: Community Management of Acute Malnutrition
NGO: Non-Governmental Organisation
GAM: Global acute malnutrition
SAM: Severe acute malnutrition
MAM: Moderate acute malnutrition
WHZ: Weight for height Z-score
